# 4-Meth­oxy-*N*-[6-methyl-2,3-dihydro-1,3-benzothia­zol-2-yl­idene]benzene­sulfonamide

**DOI:** 10.1107/S1600536807065312

**Published:** 2007-12-06

**Authors:** Gabriel Navarrete-Vázquez, Hermenegilda Moreno-Diaz, Rafael Villalobos-Molina, Samuel Estrada-Soto, Hugo Tlahuext

**Affiliations:** a Facultad de Farmacia, Universidad Autónoma del Estado de Morelos, Av. Universidad 1001 Col Chamilpa CP 62100, Cuernavaca Mor., Mexico; b Unidad de Biomedicina, FES Iztacala, Universidad Nacional Autónoma de, Mexico, Tlalnepantla, Méx. 54090, Mexico; c Centro de Investigaciones Químicas, Universidad Autónoma del Estado de Morelos. Av. Universidad 1001 Col., Chamilpa, CP 62100, Cuernavaca Mor., Mexico

## Abstract

The title compound, C_15_H_14_N_2_O_3_S_2_, is of inter­est with respect to its biological activity. The crystal structure is stabilized by inter­molecular N—H⋯N, C—H⋯O and C—H⋯π hydrogen-bonding inter­actions, as well as offset π–π inter­actions [distance between the centroids of the aryl and thiazole rings of adjacent molecules of 3.954 (2) Å].

## Related literature

For related literature, see: Su *et al.* (2006 ▶); Vicker *et al.* (2007 ▶); Siddiqui *et al.* (2007 ▶); Adams *et al.* (1996 ▶); Bernstein *et al.* (1995 ▶); Desiraju (1991 ▶); Hanton *et al.* (1992 ▶); Hunter (1994 ▶).
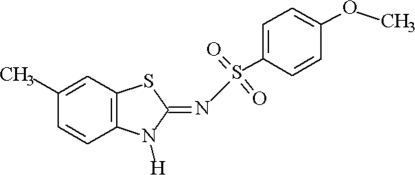


## Experimental

### 

#### Crystal data

C_15_H_14_N_2_O_3_S_2_
*M* *_r_* = 334.40
Monoclinic, 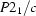

*a* = 12.0173 (15) Å
*b* = 16.211 (2) Å
*c* = 7.7377 (10) Å
β = 99.973 (2)°
*V* = 1484.6 (3) Å^3^
*Z* = 4
Mo *K*α radiation
μ = 0.37 mm^−1^
*T* = 273 (2) K
0.57 × 0.16 × 0.10 mm

#### Data collection

Bruker SMART APEX CCD area-detector diffractometer
Absorption correction: multi-scan (*SADABS*; Sheldrick, 2003 ▶) *T*_min_ = 0.816, *T*_max_ = 0.964
12102 measured reflections
2617 independent reflections
2440 reflections with *I* > 2σ(*I*)
*R*_int_ = 0.032

#### Refinement

*R*[*F* ^2^ > 2σ(*F* ^2^)] = 0.048
*wR*(*F* ^2^) = 0.117
*S* = 1.15
2617 reflections
205 parameters
H atoms treated by a mixture of independent and constrained refinement
Δρ_max_ = 0.41 e Å^−3^
Δρ_min_ = −0.20 e Å^−3^

### 

Data collection: *SMART* (Bruker, 2000 ▶); cell refinement: *SAINT-Plus-NT* (Bruker, 2000 ▶); data reduction: *SAINT-Plus-NT*; program(s) used to solve structure: *SHELXS97* (Sheldrick, 1997 ▶); program(s) used to refine structure: *SHELXL97* (Sheldrick, 1997 ▶); molecular graphics: *SHELXTL-NT* (Bruker, 2000 ▶); software used to prepare material for publication: *PLATON* (Spek, 2003 ▶) and *publCIF* (Westrip, 2008 ▶).

## Supplementary Material

Crystal structure: contains datablocks I, global. DOI: 10.1107/S1600536807065312/at2516sup1.cif

Structure factors: contains datablocks I. DOI: 10.1107/S1600536807065312/at2516Isup2.hkl

Additional supplementary materials:  crystallographic information; 3D view; checkCIF report

## Figures and Tables

**Table 1:** Hydrogen-bond geometry (Å, °)

*D*—H⋯*A*	*D*—H	H⋯*A*	*D*⋯*A*	*D*—H⋯*A*
N1—H1⋯N2^i^	0.89 (2)	2.07 (2)	2.948 (3)	169 (2)
C3—H3⋯O2^i^	0.93	2.41	3.248 (3)	150
C6—H6⋯O3^ii^	0.93	2.57	3.485 (3)	169
C9—H9⋯O1	0.93	2.51	2.894 (3)	105
C14—H14*A*⋯*Cg*3^iii^	0.96	2.76	3.433 (3)	128

Symmetry codes: (i) 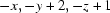; (ii) 
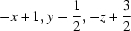
; (iii) 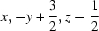. *Cg*3 is the centroid of the C8–C13 benzene ring.

**Fig. 1.**
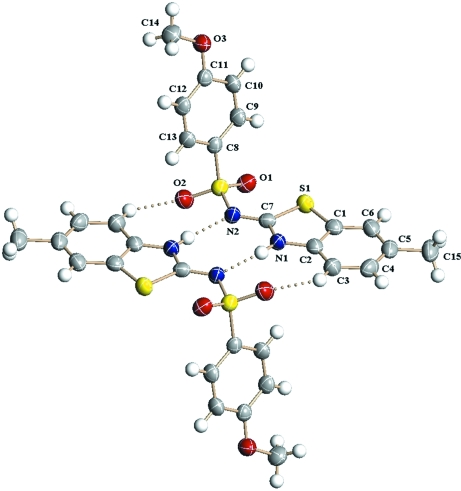
The molecular structure of (I) showing 50% probability displacement ellipsoids, H atoms are shown as small spheres of arbitrary radius and the atomic numbering. The intermolecular hydrogen bonds N—H···N and C—H···O, which are forming the R22(8) motifs are shown as dotted lines.

**Fig. 2.**
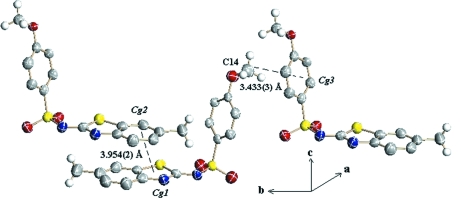
A view of the π-facial hydrogen bonds (OCH3···π) and offset π-π interactions. Dashed lines indicate the interaction between methoxy group (C14) and centroid Cg3, as well as between centroids (Cg1, Cg2).

## supplementary crystallographic information

### Comment

Benzothiazole benzenesulfonamides are selective inhibitors of 11β-hydroxysteroid dehydrogenase type 1 (11β-HSD1). They have a considerable potential use for the treatment of metabolic diseases, such as diabetes mellitus type 2 or obesity (Su *et al.*, 2006; Vicker *et al.*, 2007). This kind of compounds have also shown anticonvulsant activity (Siddiqui *et al.*, 2007).

In order to assist our knowledge about the electronic and steric requirements to shown antihyperglycemic activity, we have determined the crystal structure of the title compound, which is a new chemical entity with potential use in the treatment of diabetes.

The title compound (I) crystallizes in the centrosymmetric monoclinic space group P21/c. The crystal structure is stabilized by strong hydrogen-bonding interactions N1—H1···N2, and weak hydrogen bonding interactions C—H···O, which are forming *R*_2_^2^(8) motifs (Bernstein *et al.*, 1995) Fig. 1, Table 2. In the crystal packing there are also π-facial hydrogen bonds between the methoxy group (C14) and C8—C13 benzene ring (O—CH_3_···π) (Hanton *et al.*, 1992; Adams *et al.*, 1996, Desiraju, 1991). The distance between C14 and the ring centroid (*Cg*3) is 3.433 (3) Å (Fig. 2, Table 2).

The crystal structure is also stabilized by offset π-π interactions between two adjacent molecules, with a distance between the centroids of the C1—C6 benzene ring (*Cg*2) and (S2/C7/N1/C2/C1) benzothiazol ring (*Cg*1) of 3.954 (2) Å (Fig. 2). This interaction is favourable by effect of polarization of the sulfonamid group (Hunter, 1994).

### Experimental

To a solution of 2-amino-6-methylbenzothiazole (0.0030 mol) in dichloromethane (10 ml) were added triethylamine (1.1 eq), and a catalytic amount of dimethylaminopyridine (DMAP). After stirring at room temperature for 15 min, a solution of 4-methoxybenzenesulfonyl chloride (0.0033 mol, 1.1 eq) in 5 ml of dichloromethane was added droopingly. The reaction mixture was stirred at 313 K under nitrogen atmosphere for 6 h. After complete conversion as indicated by TLC, the solvent was removed *in vacuo*, the residue was neutralized with saturated NaHCO_3_ solution, and the aqueous layer was extracted with ethyl acetate (3 *x* 15 ml), washed with water (3 *x* 20 ml), and dried over anhydrous Na_2_SO_4_. The solvent was evaporated *in vacuo* to give a yellow solid (m.p. 534.4 K). Single crystals of (I) were obtained from acetonitrile.

### Refinement

The hydrogen H1 was located in a difference Fourier map and was refined freely. The other H atoms were constrained to the riding-model approximation [*C*—H_aryl_ = 0.93 Å, *U*_iso_(H_aryl_) = 1.2 *U*_eq_(C_aryl_); *C*—H_methyl_ = 0.96 Å, *U*_iso_(H) = 1.5 *U*_eq_(C_methyl_)].

### Crystal data

**Table:**

C_15_H_14_N_2_O_3_S_2_	*F*_000_ = 696
*M**_r_* = 334.40	*D*_x_ = 1.496 Mg m^−^^3^
Monoclinic, *P*2_1_/*c*	Melting point: 534.4 K
Hall symbol: -P 2ybc	Mo *K*α radiation λ = 0.71073 Å
*a* = 12.0173 (15) Å	Cell parameters from 2617 reflections
*b* = 16.211 (2) Å	θ = 1.7–25.0º
*c* = 7.7377 (10) Å	µ = 0.37 mm^−^^1^
β = 99.973 (2)º	*T* = 273 (2) K
*V* = 1484.6 (3) Å^3^	Rectangular prism, yellow
*Z* = 4	0.57 × 0.16 × 0.10 mm

### Data collection

**Table:**

Bruker SMART APEX CCD area-detector diffractometer	2617 independent reflections
Radiation source: fine-focus sealed tube	2440 reflections with *I* > 2σ(*I*)
Monochromator: graphite	*R*_int_ = 0.032
Detector resolution: 8.3 pixels mm^-1^	θ_max_ = 25.0º
*T* = 273(2) K	θ_min_ = 1.7º
φ and ω scans	*h* = −14→14
Absorption correction: multi-scan(SADABS; Sheldrick, 2003)	*k* = −19→18
*T*_min_ = 0.816, *T*_max_ = 0.964	*l* = −9→9
12102 measured reflections	

### Refinement

**Table:**

Refinement on *F*^2^	Secondary atom site location: difference Fourier map
Least-squares matrix: full	Hydrogen site location: inferred from neighbouring sites
*R*[*F*^2^ > 2σ(*F*^2^)] = 0.048	H atoms treated by a mixture of independent and constrained refinement
*wR*(*F*^2^) = 0.117	*w* = 1/[σ^2^(*F*_o_^2^) + (0.0504*P*)^2^ + 0.9002*P*] where *P* = (*F*_o_^2^ + 2*F*_c_^2^)/3
*S* = 1.15	(Δ/σ)_max_ = 0.001
2617 reflections	Δρ_max_ = 0.41 e Å^−^^3^
205 parameters	Δρ_min_ = −0.19 e Å^−^^3^
Primary atom site location: structure-invariant direct methods	Extinction correction: none

### Special details

**Table:**

Geometry. All e.s.d.'s (except the e.s.d. in the dihedral angle between two l.s. planes) are estimated using the full covariance matrix. The cell e.s.d.'s are taken into account individually in the estimation of e.s.d.'s in distances, angles and torsion angles; correlations between e.s.d.'s in cell parameters are only used when they are defined by crystal symmetry. An approximate (isotropic) treatment of cell e.s.d.'s is used for estimating e.s.d.'s involving l.s. planes.
Refinement. Refinement of *F*^2^ against ALL reflections. The weighted *R*-factor *wR* and goodness of fit *S* are based on *F*^2^, conventional *R*-factors *R* are based on *F*, with *F* set to zero for negative *F*^2^. The threshold expression of *F*^2^ > σ(*F*^2^) is used only for calculating *R*-factors(gt) *etc*. and is not relevant to the choice of reflections for refinement. *R*-factors based on *F*^2^ are statistically about twice as large as those based on *F*, and *R*- factors based on ALL data will be even larger.

### Fractional atomic coordinates and isotropic or equivalent isotropic displacement parameters (Å^2^)

**Table:**

	*x*	*y*	*z*	*U*_iso_*/*U*_eq_	
C1	0.1795 (2)	0.78238 (17)	0.4365 (3)	0.0450 (6)	
C2	0.0744 (2)	0.80223 (16)	0.4774 (3)	0.0422 (6)	
C3	−0.0003 (2)	0.74059 (17)	0.5022 (4)	0.0512 (7)	
H3	−0.0704	0.7531	0.5307	0.061*	
C4	0.0318 (3)	0.66009 (18)	0.4836 (4)	0.0556 (7)	
H4	−0.0185	0.6183	0.4993	0.067*	
C5	0.1359 (3)	0.63832 (18)	0.4425 (4)	0.0524 (7)	
C6	0.2108 (2)	0.70089 (18)	0.4188 (4)	0.0498 (7)	
H6	0.2812	0.6883	0.3913	0.060*	
C7	0.1455 (2)	0.93390 (16)	0.4535 (3)	0.0402 (6)	
C8	0.3207 (2)	1.09049 (15)	0.6440 (3)	0.0385 (6)	
C9	0.4280 (2)	1.05758 (17)	0.6913 (4)	0.0455 (6)	
H9	0.4576	1.0234	0.6139	0.055*	
C10	0.4905 (2)	1.07537 (17)	0.8519 (4)	0.0469 (6)	
H10	0.5630	1.0540	0.8824	0.056*	
C11	0.4465 (2)	1.12510 (15)	0.9704 (3)	0.0408 (6)	
C12	0.3377 (2)	1.15595 (17)	0.9262 (4)	0.0451 (6)	
H12	0.3068	1.1877	1.0061	0.054*	
C13	0.2755 (2)	1.13914 (16)	0.7626 (4)	0.0440 (6)	
H13	0.2030	1.1605	0.7316	0.053*	
C14	0.4823 (3)	1.1987 (2)	1.2418 (4)	0.0591 (8)	
H14A	0.4687	1.2510	1.1838	0.089*	
H14B	0.5405	1.2046	1.3429	0.089*	
H14C	0.4141	1.1798	1.2778	0.089*	
C15	0.1682 (3)	0.54964 (19)	0.4239 (4)	0.0669 (9)	
H15A	0.1849	0.5246	0.5379	0.100*	
H15B	0.2337	0.5469	0.3686	0.100*	
H15C	0.1068	0.5208	0.3534	0.100*	
H1	−0.003 (2)	0.9100 (16)	0.509 (3)	0.046 (8)*	
N1	0.05952 (18)	0.88679 (13)	0.4860 (3)	0.0426 (5)	
N2	0.13799 (17)	1.01569 (13)	0.4570 (3)	0.0434 (5)	
O1	0.31808 (16)	1.03121 (12)	0.3323 (2)	0.0513 (5)	
O2	0.19594 (16)	1.14977 (12)	0.3663 (3)	0.0563 (5)	
O3	0.51706 (16)	1.14040 (12)	1.1242 (3)	0.0541 (5)	
S1	0.24400 (5)	1.07274 (4)	0.43153 (8)	0.0417 (2)	
S2	0.25626 (5)	0.87158 (5)	0.40816 (9)	0.0480 (2)	

### Atomic displacement parameters (Å^2^)

**Table:**

	*U*^11^	*U*^22^	*U*^33^	*U*^12^	*U*^13^	*U*^23^
C1	0.0430 (14)	0.0497 (16)	0.0401 (14)	0.0028 (12)	0.0015 (11)	−0.0023 (12)
C2	0.0412 (13)	0.0448 (15)	0.0388 (13)	0.0035 (12)	0.0014 (11)	−0.0044 (11)
C3	0.0462 (15)	0.0451 (16)	0.0613 (17)	−0.0012 (13)	0.0062 (13)	−0.0020 (14)
C4	0.0613 (18)	0.0438 (16)	0.0585 (18)	−0.0095 (14)	0.0017 (14)	0.0016 (13)
C5	0.0669 (18)	0.0441 (16)	0.0414 (15)	0.0084 (14)	−0.0041 (13)	−0.0028 (12)
C6	0.0512 (16)	0.0512 (17)	0.0457 (15)	0.0115 (13)	0.0042 (12)	−0.0017 (12)
C7	0.0334 (12)	0.0466 (15)	0.0395 (13)	−0.0003 (11)	0.0039 (10)	−0.0051 (11)
C8	0.0346 (12)	0.0335 (13)	0.0491 (14)	−0.0050 (10)	0.0119 (11)	−0.0019 (11)
C9	0.0432 (14)	0.0447 (15)	0.0512 (15)	0.0082 (12)	0.0150 (12)	−0.0060 (12)
C10	0.0392 (14)	0.0462 (16)	0.0561 (16)	0.0104 (12)	0.0106 (12)	−0.0031 (12)
C11	0.0416 (13)	0.0344 (13)	0.0472 (14)	−0.0011 (11)	0.0097 (11)	0.0001 (11)
C12	0.0418 (14)	0.0442 (15)	0.0526 (16)	0.0034 (12)	0.0173 (12)	−0.0077 (12)
C13	0.0313 (12)	0.0462 (15)	0.0557 (16)	0.0038 (11)	0.0108 (11)	−0.0052 (12)
C14	0.0651 (19)	0.0595 (19)	0.0521 (17)	0.0015 (15)	0.0086 (14)	−0.0137 (14)
C15	0.090 (2)	0.0467 (18)	0.0602 (19)	0.0139 (17)	0.0018 (17)	−0.0001 (15)
N1	0.0339 (11)	0.0405 (13)	0.0538 (13)	0.0021 (10)	0.0084 (10)	−0.0054 (10)
N2	0.0348 (11)	0.0393 (12)	0.0568 (13)	−0.0015 (9)	0.0098 (10)	−0.0064 (10)
O1	0.0471 (10)	0.0587 (13)	0.0518 (11)	−0.0061 (9)	0.0184 (9)	−0.0104 (9)
O2	0.0562 (12)	0.0495 (12)	0.0611 (12)	0.0012 (9)	0.0046 (10)	0.0077 (9)
O3	0.0507 (11)	0.0564 (12)	0.0532 (11)	0.0081 (9)	0.0031 (9)	−0.0098 (9)
S1	0.0377 (3)	0.0418 (4)	0.0469 (4)	−0.0029 (3)	0.0109 (3)	−0.0028 (3)
S2	0.0389 (4)	0.0487 (4)	0.0582 (4)	0.0048 (3)	0.0137 (3)	−0.0031 (3)

### Geometric parameters (Å, °)

**Table:**

C1—C6	1.387 (4)	C9—H9	0.9300
C1—C2	1.391 (4)	C10—C11	1.392 (4)
C1—S2	1.749 (3)	C10—H10	0.9300
C2—C3	1.378 (4)	C11—O3	1.359 (3)
C2—N1	1.385 (3)	C11—C12	1.387 (4)
C3—C4	1.375 (4)	C12—C13	1.381 (4)
C3—H3	0.9300	C12—H12	0.9300
C4—C5	1.388 (4)	C13—H13	0.9300
C4—H4	0.9300	C14—O3	1.424 (3)
C5—C6	1.389 (4)	C14—H14A	0.9600
C5—C15	1.502 (4)	C14—H14B	0.9600
C6—H6	0.9300	C14—H14C	0.9600
C7—N2	1.330 (3)	C15—H15A	0.9600
C7—N1	1.343 (3)	C15—H15B	0.9600
C7—S2	1.754 (3)	C15—H15C	0.9600
C8—C9	1.385 (4)	N1—H1	0.89 (3)
C8—C13	1.390 (3)	N2—S1	1.614 (2)
C8—S1	1.764 (3)	O1—S1	1.4394 (19)
C9—C10	1.367 (4)	O2—S1	1.431 (2)
			
C6—C1—C2	121.0 (3)	C12—C11—C10	119.7 (2)
C6—C1—S2	128.1 (2)	C13—C12—C11	119.5 (2)
C2—C1—S2	110.9 (2)	C13—C12—H12	120.2
C3—C2—N1	128.2 (2)	C11—C12—H12	120.2
C3—C2—C1	120.1 (3)	C12—C13—C8	120.4 (2)
N1—C2—C1	111.7 (2)	C12—C13—H13	119.8
C4—C3—C2	118.2 (3)	C8—C13—H13	119.8
C4—C3—H3	120.9	O3—C14—H14A	109.5
C2—C3—H3	120.9	O3—C14—H14B	109.5
C3—C4—C5	123.0 (3)	H14A—C14—H14B	109.5
C3—C4—H4	118.5	O3—C14—H14C	109.5
C5—C4—H4	118.5	H14A—C14—H14C	109.5
C4—C5—C6	118.3 (3)	H14B—C14—H14C	109.5
C4—C5—C15	121.6 (3)	C5—C15—H15A	109.5
C6—C5—C15	120.1 (3)	C5—C15—H15B	109.5
C1—C6—C5	119.3 (3)	H15A—C15—H15B	109.5
C1—C6—H6	120.4	C5—C15—H15C	109.5
C5—C6—H6	120.4	H15A—C15—H15C	109.5
N2—C7—N1	120.4 (2)	H15B—C15—H15C	109.5
N2—C7—S2	129.42 (19)	C7—N1—C2	116.3 (2)
N1—C7—S2	110.18 (19)	C7—N1—H1	120.3 (17)
C9—C8—C13	119.8 (2)	C2—N1—H1	123.4 (17)
C9—C8—S1	119.73 (19)	C7—N2—S1	120.74 (17)
C13—C8—S1	120.45 (19)	C11—O3—C14	118.2 (2)
C10—C9—C8	119.9 (2)	O2—S1—O1	117.99 (12)
C10—C9—H9	120.1	O2—S1—N2	105.33 (12)
C8—C9—H9	120.1	O1—S1—N2	111.79 (11)
C9—C10—C11	120.7 (2)	O2—S1—C8	107.49 (12)
C9—C10—H10	119.7	O1—S1—C8	107.55 (12)
C11—C10—H10	119.7	N2—S1—C8	106.02 (12)
O3—C11—C12	124.7 (2)	C1—S2—C7	90.91 (12)
O3—C11—C10	115.6 (2)		
			
C6—C1—C2—C3	0.4 (4)	S1—C8—C13—C12	−177.1 (2)
S2—C1—C2—C3	178.9 (2)	N2—C7—N1—C2	179.3 (2)
C6—C1—C2—N1	−179.0 (2)	S2—C7—N1—C2	−0.1 (3)
S2—C1—C2—N1	−0.5 (3)	C3—C2—N1—C7	−179.0 (3)
N1—C2—C3—C4	178.7 (3)	C1—C2—N1—C7	0.4 (3)
C1—C2—C3—C4	−0.6 (4)	N1—C7—N2—S1	175.70 (19)
C2—C3—C4—C5	0.5 (4)	S2—C7—N2—S1	−5.1 (3)
C3—C4—C5—C6	−0.1 (4)	C12—C11—O3—C14	−6.8 (4)
C3—C4—C5—C15	179.8 (3)	C10—C11—O3—C14	172.5 (2)
C2—C1—C6—C5	−0.1 (4)	C7—N2—S1—O2	155.3 (2)
S2—C1—C6—C5	−178.3 (2)	C7—N2—S1—O1	25.9 (2)
C4—C5—C6—C1	−0.1 (4)	C7—N2—S1—C8	−91.0 (2)
C15—C5—C6—C1	−180.0 (3)	C9—C8—S1—O2	−134.5 (2)
C13—C8—C9—C10	−2.2 (4)	C13—C8—S1—O2	43.7 (2)
S1—C8—C9—C10	176.0 (2)	C9—C8—S1—O1	−6.5 (2)
C8—C9—C10—C11	1.2 (4)	C13—C8—S1—O1	171.7 (2)
C9—C10—C11—O3	−178.4 (2)	C9—C8—S1—N2	113.2 (2)
C9—C10—C11—C12	1.0 (4)	C13—C8—S1—N2	−68.6 (2)
O3—C11—C12—C13	177.1 (2)	C6—C1—S2—C7	178.7 (3)
C10—C11—C12—C13	−2.1 (4)	C2—C1—S2—C7	0.4 (2)
C11—C12—C13—C8	1.1 (4)	N2—C7—S2—C1	−179.5 (2)
C9—C8—C13—C12	1.1 (4)	N1—C7—S2—C1	−0.15 (19)

### Hydrogen-bond geometry (Å, °)

**Table:**

*D*—H···*A*	*D*—H	H···*A*	*D*···*A*	*D*—H···*A*
N1—H1···N2^i^	0.89 (2)	2.07 (2)	2.948 (3)	169 (2)
C3—H3···O2^i^	0.93	2.41	3.248 (3)	150
C6—H6···O3^ii^	0.93	2.57	3.485 (3)	169
C9—H9···O1	0.93	2.51	2.894 (3)	105
C14—H14A···Cg3^iii^	0.93	2.76	3.433 (3)	128

Symmetry codes: (i) −*x*, −*y*+2, −*z*+1; (ii) −*x*+1, *y*−1/2, −*z*+3/2; (iii) *x*, −*y*+3/2, *z*−1/2.
